# EGFR-expression in primary urinary bladder cancer and corresponding metastases and the relation to HER2-expression. On the possibility to target these receptors with radionuclides

**DOI:** 10.2478/raon-2014-0015

**Published:** 2015-03-03

**Authors:** Jörgen Carlsson, Kenneth Wester, Manuel De La Torre, Per-Uno Malmström, Truls Gårdmark

**Affiliations:** 1 Department of Radiology, Oncology and Radiation Science, Biomedical Radiation Sciences, Rudbeck Laboratory, Uppsala University, Uppsala, Sweden; 2 Department of Immunology, Genetics and Pathology, Molecular and Morphological Pathology, Rudbeck Laboratory, Uppsala University Hospital, Uppsala, Sweden; 3 Department of Surgical Sciences, Urology, Uppsala University Hospital, Uppsala, Sweden; 4 Department of Clinical Sciences, General Surgery and Urology, Karolinska Institute at Danderyd Hospital, Stockholm, Sweden

**Keywords:** EGFR, HER2, radionuclides, resistance, urinary bladder cancer metastases

## Abstract

**Background:**

There is limited effect of tyrosine kinase inhibitors or “naked” antibodies binding EGFR or HER2 for therapy of metastasized urinary bladder cancer and these methods are therefore not routinely used. Targeting radio-nuclides to the extracellular domain of the receptors is potentially a better possibility.

**Methods:**

EGFR- and HER2-expression was analyzed for primary tumors and corresponding metastases from 72 patients using immunohistochemistry and the internationally recommended HercepTest. Intracellular mutations were not analyzed since only the receptors were considered as targets and intracellular abnormalities should have minor effect on radiation dose.

**Results:**

EGFR was positive in 71% of the primary tumors and 69% of corresponding metastases. Local and distant metastases were EGFR-positive in 75% and 66% of the cases, respectively. The expression frequency of HER2 in related lesions was slightly higher (data from previous study). The EGFR-positive tumors expressed EGFR in metastases in 86% of the cases. The co-expression of EGFR and HER2 was 57% for tumors and 53% for metastases. Only 3% and 10% of the lesions were negative for both receptors in tumors and metastases, respectively. Thus, targeting these receptors with radionuclides might be applied for most patients.

**Conclusions:**

At least one of the EGFR- or HER2-receptors was present in most cases and co-expressed in more than half the cases. It is therefore interesting to deliver radionuclides for whole-body receptor-analysis, dosimetry and therapy. This can hopefully compensate for resistance to other therapies and more patients can hopefully be treated with curative instead of palliative intention.

## Introduction

Biological resistance to both EGFR- and HER2-targeted therapies, due to mutations in for example PI3K/AKT, Ras/Raf/Mek/Erk or other intracellular signal pathways has been observed for many types of cancer.^1^–^4^ Urinary bladder cancer is at present not generally considered for therapy with EGFR-or HER2-binding agents such as tyrosine kinase inhibitors and “naked” antibodies (*e.g*. trastuzumab or cetuximab). Evidence for therapy efficacy of such agents in urinary bladder cancer is lacking and it has been claimed that there might, in several cases, be resistance.^5^–^8^ It might therefore be, as an alternative to tyrosine kinase inhibitors and “naked” antibodies, beneficial to target the extracellular domains of EGFR and/or HER2 in metastatic urinary bladder cancer patients with molecules that deliver suitable radionuclides not only for whole body receptor mapping and dosimetry but also for radionuclide therapy. Examples of radionuclides for these purposes are given in the Discussion.

Therapy with radionuclides is of interest since induced resistance to effects of radiation is not a major problem in cancer therapy. The radionuclides can be delivered to cancer cells with various types of molecules, *e.g*. antibodies, antibody fragments and smaller proteins such as affibody molecules and also with peptides.^9^–^12^ The application of radionuclide labeled molecules for EGFR- and/or HER2-targeted therapy has so far, to the knowledge of the authors, not been clinically applied for therapy of metastatic urinary bladder cancer. If this is tried, the strategy is that the radionuclides can kill cancer cells independent of possible intracellular mutations. This is also why we decided to neither analyze mutations in the intracellular signal pathways nor gene amplifications.

EGFR and HER2 belong to the type 1 tyrosine kinase receptor family consisting of four related receptors, forming dimers with each other, and are important for growth of various cancers.^13^ Several agents binding to EGFR and HER2 aimed to interfere with intracellular downstream signaling, and give therapy effects, are developed or are under development.^14^–^18^ Binders to the other receptors in the EGFR-family, *i.e*. HER3 and HER4, has so far not been introduced for clinical applications so we focus only on EGFR and HER2 in this study.

The worldwide incidence of urinary bladder cancer is high with 350–400.000 new cases per year and the incidence is high also in Europe.^19^–^21^ Furthermore, approximately one third of all urinary bladder cancers are, at the time of diagnosis, growing invasive through the bladder wall and can form metastases which often are growing in regional (local) lymph nodes and in several distant organs, especially lung, liver and skeleton.^22^ External radiotherapy and surgery are treatment modalities for the localized tumors. Chemotherapy and tyrosine kinase inhibitors are applied for therapy of the disseminated tumors but such therapy is in most cases not curative.^5^,^6^,^22^ Thus, other treatment modalities, *e.g*. receptor targeted radionuclide therapy is of interest to exploit. We analyzed and discussed in this article whether EGFR and HER2 are expressed with such high frequencies that targeted radionuclide therapy might be a possibility and an alternative or complement to other modalities in the treatment of metastatic urinary bladder cancers.

## Materials and methods

### Tissue samples

The study included 72 patients with metastatic urinary bladder carcinoma, where tissue samples from both primary tumors and metastases were available. The study was approved by the institutional review board. In the previous publication 90 patients were analysed^22^ but samples were not available for 18 of the patients because the paraffin blocks were previously sectioned so much that no tissue of value could be found. The primary treatment was transurethral resection in 61 (85%) cases and cystectomy in 11 (15%) cases. Patient, tumor and metastasis characteristics are shown in Table 1. All samples were fixated in 10% buffered formalin (4% formaldehyde), paraffin embedded according to standard procedures at our laboratory and sections, 4-μm thick, were then cut and deparaffinized in xylene and hydrated through graded concentrations of ethanol to distilled water.^23^

### EGFR-immunohistochemistry

EGFR was assessed by immunohistochemistry, IHC, using a streptavidin-biotin complex technique as previously described.^23^,^24^ Endogenous peroxidase was blocked in 0.3% H_2_O_2_ in PBS for 20 min. Then, antigen retrieval was done in 0.05% protease K (Code no. S3020, Dako, Glostrup, Denmark) in PBS for 10 min at room temperature. The tissue sections were preincubated in PBS for 10 min and then the primary mouse monoclonal antibody (clone 31G7, Zymed labs, South San Francisco, CA, USA) directed against the EGF receptor, diluted 1:100, was added and the sections incubated overnight at 4°C. The secondary biotinylated antibody (goat anti-mouse, Dako, Glostrup, Denmark) and the peroxidase-labeled streptavidin-biotin complex (Dako, Glostrup, Denmark), diluted 1:200, were then added and the samples incubated for 30 min at room temperature. All sections were developed in 0.05% diamino benzidine (Sigma, St Louis, MO, USA) for 5 min and counterstained in Harris haematoxylin (Sigma, St Louis, MO, USA). Finally, the sections were dehydrated through graded alcohol to xylene and mounted in organic mounting medium (Pertex®, Histolab, Gothenburg, Sweden).

### HER2-immunohistochemistry

The IHC staining was made as previously described.^22^,^24^ The sections were incubated in methanol and hydrogen peroxide for 30 min to quench endogenous peroxidase. Antigen retrieval was done in water bath at 95–98°C, pH 6 for 40 minutes. Thereafter the sections were cooled at room temperature and then washed in distilled water. Staining was performed using the Elite ABC Kit (Vectastain, Vector Laboratories, Burlingame, CA, USA). First blocking serum was applied for 15 min and followed by incubation with polyclonal rabbit anti-human c-erbB-2 oncoprotein (code No. A 0485, Dako, Glostrup, Denmark) diluted 1:350. The samples were then incubated with the biotinylated secondary antibody and visualized using the peroxidase substrate 3-amino-9-ethyl-carbazole (AEC) (Sigma A-5754, St Louis, MO, USA) as chromogen. Finally, the sections were counterstained with Mayer’s haematoxylin and mounted with Aquamount (BDH Ltd, Poole, UK).

### Evaluation of immunohistochemistry

Evaluation of the IHC staining was performed by four of the authors. Authors JC and MT evaluated the expression in this study. TG, MT and KW evaluated the HER2 expression in the previously published study.^22^

The cellular membrane expression pattern of EGFR is similar to that of HER2. EGFR expression was therefore evaluated using the same scoring criterion as for HER2. Briefly, the EGFR- and HER2-expressions were scored using the HercepTest criterion based on a scale where 0 corresponded to tumor cells that were completely negative, 1+ corresponded to faint perceptible staining of the tumor cell membranes, 2+ corresponded to moderate staining of the entire tumor cell membranes and 3+ was strong circumferential staining of the entire tumor cell membranes creating a fishnet pattern. The Canadian and the DAKO HercepTest guidelines^25^ were applied, which require ≥10% of the tumor cells to be stained. The HER2 data, as published by Gårdmark *et al*.^22^, also reported on the criterion that ≥2/3 of the tumor cells must be HER2-stained to score a sample HER2-positive. However, in the present study we only considered data applying the ≥10% criterion since only the internationally recommended HercepTest scoring^25^ made the same way as for breast cancer is accepted at our hospital.

Cytoplasmic staining was considered non-specific and was not included in the scoring. As positive controls we used positive tissue sections; epithelial samples from skin for EGFR-positivity and HER2-positive breast cancer samples complemented with HER2-positive sections from DAKO. All positive controls were stained with the same protocol as applied for the urinary bladder cancer sections. As negative controls we used normal tissues which are expected not to express the receptors such as connective tissue, blood cells and lymphocytes seen in the same sections as the tumor cells. In the metastases sections we used lymphocytes, connective tissue and the surrounding capsule of the lymph nodes as negative internal controls. Thus, all tissues used as negative controls were stained with the same protocol as the urinary bladder cancers since they were in the same sections.

### Positive versus negative receptor expression

Scores 2+ and 3+ were considered receptor positive and scores 0 and 1+ receptor negative. This a “crude” rule but gives an indication of which patients that are candidates for nuclear medicine based receptor analysis and thereafter considered suitable for radionuclide therapy.

## Results

### Expression of EGFR

**FIGURE 1.**
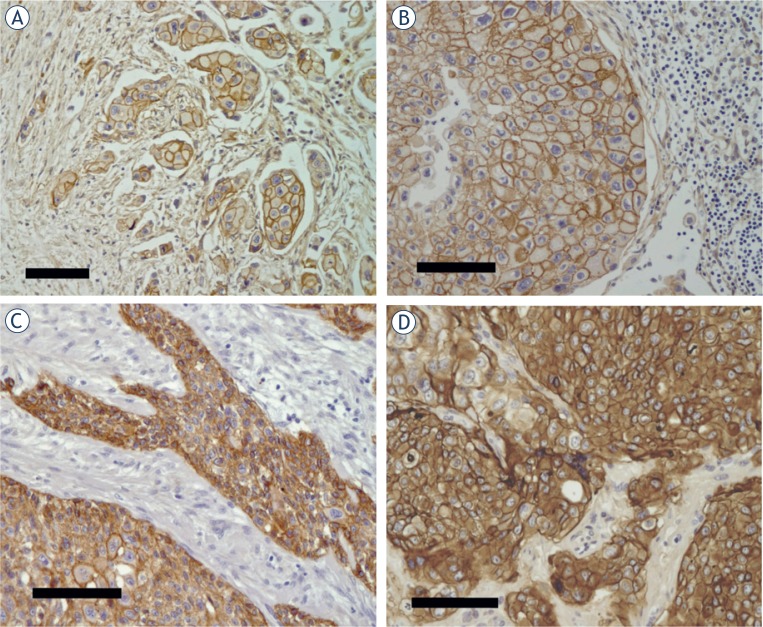
Examples of immunohistochemical EGFR-staining (brown) of samples from metastasized urinary bladder cancers. **(A)** Primary tumor. **(B)** Regional (local) lymph node metastasis (from the same patient as in A). Note the large number of lymphocytes (small blue haematoxylin stained nuclei to the right). **(C)** Primary tumor. **(D)** Colon metastasis (classified as distant). All samples were scored 3+ and EGFR-positive. All bars correspond to 100μm.

The EGFR determinations are shown in Table 2. EGFR was positive in 51/72 (71%) of the primary tumors and 50/72 (69%) of the corresponding metastases. The regional (local) and the distant metastases were EGFR-positive in 21/28 (75%) and 29/44 (66%) of the cases, respectively. Considering only the EGFR-positive primary tumors (n=51) the corresponding metastases were EGFR-positive in 44/51 (86%) of the cases. Examples of EGFR-positive tumor cells are shown in Figure 1.

There was good agreement between the expression frequencies in the primary tumors and the corresponding lymph node metastases in the majority of cases. Totally, 13 changes were observed, *i.e*. 6 upregulations and 7 downregulations, in the metastases in relation to the primary tumors and 11 of these changes were in the distant metastases (Table 2). Nine of the regional (local) lymph nodes metastases were characterized as sentinel nodes and 5 of these were EGFR-positive in both the primary tumors and the metastasis while 3 were negative in both. One sentinel node metastasis was negative while the corresponding primary tumor was positive.

### Expression of HER2

The values for HER2 positivity in our article from 2005 were 79% for primary tumors and 62% for metastases when ≥2/3 of the tumor cells had to be HER2-stained and the staining should be as intense as for breast cancer sections.^22^ The internationally recommended DAKO procedure^25^ which we used this time gave 60/72 (83%) and 53/72 (74%) HER2-positive cases in the analyzed primary tumors and metastases, respectively. The reasons for the different results are discussed below. Eight out of the 9 sentinel nodes were HER2-positive in both the primary tumors and the metastasis and one sentinel node metastasis was HER2 negative while the corresponding primary tumor was positive.

### Co-expression of EGFR and HER2

Table 3 shows the relation between EGFR expression and HER2 expression frequencies in primary tumors and metastases. The co-expression of EGFR and HER2 was 41/72 (57%) for the primary tumors and 38/72 (53%) for the metastases. Only 2/72 (3%) of the patients were negative for both receptors in the primary tumors and in 7/72 (10%) of the metastases. Table 4 shows a summary of relations between positive (2+ and 3+) and negative (0 and 1+) expressions of EGFR and between EGFR and HER2 in primary tumors and metastases.

## Discussion

At least one of the receptors, EGFR or HER2, was positive in nearly all cases and co-expression was seen in more than half of the cases and this was valid for both primary tumors and metastases. With such high expression frequencies the number of patients that can be considered for radionuclide based receptor mapping (imaging), dosimetry and therapy is high. Furthermore, targeting of both receptors in the same patient might, in many cases, be a possibility to deliver increased amounts of radionuclides, *i.e*. give higher radiation doses to the tumor cells.

Radionuclide therapy is of special interest when there is “biological resistance” to EGFR- and/or HER2-targeted therapies probably because of intracellular signal pathway mutations^1^–^4^ and such resistance, or at least lack of therapy efficacy, has been reported for urinary bladder cancer.^5^–^8^ The receptors themselves, and not intracellular mutations and gene amplifications, are of main interest in these cases.

It is of methodological interest that discrepancies between gene amplification and receptor expression has actually been reported from studies of urinary bladder cancer.^26^–^30^ However, since our primary goal was to consider the receptors as targets we focused only on IHC analyses giving receptor expression frequencies. The IHC results indicate which patients that might benefit from EGFR and/or HER2 targeted radionuclide therapy. The limitation is that the samples analyzed with IHC might neither be representative for the whole volume of tumors and metastases and that differences in expression between metastases in the same patient might exist.

Furthermore, IHC analyses cannot be a basis for radionuclide dosimetry and such calculations were of course beyond the scope of this study. Reliable information related to the spatial distribution of receptor expression and dosimetry for each patient, and possibly also for prediction of therapy results, require a well functioning whole body, non-invasive nuclear medicine based method as suggested for other types of tumors.^31^–^35^ However, the high frequencies of expression of both EGFR and HER2 in primary tumors and metastases in our study indicate that many, maybe most, urinary bladder cancers are of interest for targeting with molecules that deliver radionuclides to these receptors.

It is often claimed that positivity for EGFR and HER2 receptors indicate aggressive tumor cells and bad survival prognosis^26^,^36^–^42^ and, if so, the most aggressive cells are attacked with the radiation. The expression frequencies in our study are high in comparison to some other studies and this is probably because only patients with verified metastatic growth were considered and their primary tumors were mainly of high grade.

The expression frequency of EGFR in metastatic urinary bladder cancer has in several articles been reported to be in the range 40–100% for both primary tumors and metastases^23^,^43^–^45^ that is high enough to make EGFR an interesting target for systemic treatment. However, EGFR is also expressed in various normal tissues and for successful therapy it is required that EGFR is strongly expressed in the tumor cells, as shown in the examples in Figure 1. Favorable tumor to blood and tumor to normal tissue quotients of delivered radioactivity are required to get PET or SPECT images of high quality and this is of course also a requirement for radio-nuclide therapy. It has recently been reported that delivery molecules with low specific radioactivity (the injection solution containing a large fraction non-labeled targeting molecules) and/or delivery molecules giving fast body clearance can give good image quality.^46^,^47^,^48^ This aspect is especially important for targeting of EGFR which is expressed in various normal tissues.^49^, ^50^

The tendency towards lower HER2-expression frequencies with increasing “distance” from the primary tumor as previously indicated^22^ was in our study not present considering the EGFR-expression frequencies.

The expression frequency of HER2 has also been reported to often be high and vary a lot, *i.e*. in the range 25-100% in primary tumors^22^,^23^,^37^,^42^,^51^ and 40–70% in corresponding metastases. 22,23,38,42,52 It has been indicated that most HER2-positive metastases were from HER2-positive primary tumors.^22^,^23^,^42^ HER2 is much less expressed in normal tissues than EGFR, especially in liver, reproductive organs, the digestive tract and bile ducts and various epithelial tissues.^53^,^54^ Thus, HER2 is an interesting target for therapy with radionuclides since the uptake in normal organs is expected to be low. This also supports the possibility to combine EGFR-targeted therapy with HER2-targeted therapy.

The large differences in reported receptor expression frequencies between different studies are probably due to different patient inclusion criteria and etiological differences but differences in receptor scoring methods and IHC retrieval techniques might also be explanations. The values for HER2 positivity in our 2005 article on urinary bladder cancer^22^ were also different from the values in the present article. The reason for the difference between our investigations is unclear but improved retrieval and staining procedures can at least be part of the explanation. We previously also had a criteria that staining intensity should be comparable to that often seen in breast cancer stainings. We do not apply that criteria anymore and instead only use the internationally recommended HercepTest staining and scoring system, i.e. the DAKO procedure.^25^

Suitable molecules for delivery of radionuclides are antibodies, antibody fragments, affibody molecules and small molecules such as peptides.^9^–^12^ Bi-functional antibodies or affibody molecules, with capacity to bind two receptors at the same time, is also a possible approach for radionuclide based imaging and therapy. Co-expression may be associated with high-grade malignancy^15^,^16^ and “double targeting” can hopefully also increase targeting specificity.

Radionuclides suitable for therapy are β-emitters (e.g. ^67^Cu, ^90^Y, ^131^I, ^177^Lu, ^186^Re, ^188^Re) and α-emitters (e.g. ^211^At, ^212^Bi, ^213^Bi, ^225^Ac, ^227^Th). The requirement is of course that relevant labeling methods are available that not significantly disturb receptor binding. For receptor mapping (imaging) and dosimetry, radionuclides such as ^99m^Tc, ^111^In and ^123^I for gamma cameras (including SPECT) or ^18^F, ^64^Cu, ^68^Ga, ^76^Br, ^86^Y, ^89^Zr and ^124^I for PET cameras can be applied when relevant labeling methods are at hand.^9^–^12^,^43^

The α- and β-particle emitters can locally give rather homogeneous dose distributions since the radiation from the targeted cells can give therapeutic effects also on non-targeted tumor cells.^12^ There are also examples of interesting toxins that are candidates for receptor mediated delivery to tumor cells 55,56 but these are not discussed in this article.

Targeted radionuclide therapy has been available for many years but few methods are routinely used on a large scale. However, during the past decades promising clinical and preclinical research have been made with therapy using radiolabeled antibodies for therapy of lymphomas^57^,^58^ (*e.g*.anti-CD20 antibodies such as ^90^Y-Zevalin) and radiolabeled peptides for therapy of neuroendocrine tumors^59^,^60^ (*e.g*. somatostatin analogs such as ^177^Lu-Octreotate). There is potential for more and improved clinical studies since preclinical research and early clinical studies have given interesting results and there is also an intensive search for new targeting agents with advantageous pharmacokinetics and biodistributions.^11^,^31^–^34^,^61^–^67^ Note that promising results with radionuclide therapy of lymphomas^57^,^58^ has been achieved although the tumor cells are spread throughout large parts of the patients. This indicates that targeted radionuclide therapy can also be of value for treatment of disseminated solid tumors.

The novelty of our previous article published 2005 was to show the high percentage of HER2 expression in urinary bladder cancers and to indicate a possible value of HER2-targeted therapy (with for example lapatinib and/or trastuzumab).^22^ However, since then there has been reports on resistance of urinary bladder cancer for both EGFR-and HER2-targeting agents (e.g. cetuximab, trastuzumab, lapatinib).^5^–^8^ The novelty of the present article is to suggest targeting of the receptors with agents^9^–^12^ that deliver radionuclides of therapy interest.

In conclusion, it is of interest to try to target EGFR and/or HER2 for radionuclide based therapy of disseminated urinary bladder cancers to decrease the influence of resistance to other forms of therapy. This might improve therapy effectiveness and hopefully allow that more patients can be treated with curative instead of palliative intention.

## Figures and Tables

**TABLE 1.** Characteristics of the urinary bladder cancers and patients with available EGFR and HER2 data for both primary tumors and corresponding metastases (n=72)

**Characteristics**	**Number and (%) patients**
***Gender***	

Male	55 (≈76%)
Female	17 (≈24%)

***Primary tumor characteristics***	

Histological grade II^*^	8 (≈11%)
Histological grade III^*^	64 (≈89%)

***Metastatic locations***	

*Lymph node metastases*	
Regional (local) lymph nodes	28 (≈39%)
Distant lymph nodes	7 (≈10%)
*Non-lymph node metastases*	
Liver	6 (≈8%)
Lung	1 (≈1%)
Skeleton	3 (≈4%)
Intestinal	10 (≈14%)
Prostate	5 (≈7%)
Vagina	1 (≈1%)
Other	11 (≈15%)

The mean age of the patients at diagnosis was ≈ 66 years (span 35–87). The time from diagnosis of the primary tumor to sampling of metastases was on the average ≈ 10 months (span 0–82) (Gårdmark et al [22]).

* WHO 1977.

**TABLE 2.** EGFR-scores for all analyzed primary urinary bladder tumors and corresponding metastases (upper part), regional (local) lymph node metastases (middle part) and distant metastases and non-lymph node metastases (lower part)

**Primary tumors EGFR-scores**	**All metastases, EGFR-scores (n=72)**			

	**0**	**1+**	**2+**	**3+**

0	6	0	2	0
1+	2	7	4	0
2+	0	4	12	2
3+	2	1	5	25

**Primary tumors EGFR-scores**	**Regional (local) lymph node metastases, EGFR-scores (n=28)**			

	**0**	**1+**	**2+**	**3+**

0	3	0	0	0
1+	1	1	0	0
2+	0	2	3	2
3+	0	0	3	13

**Primary tumors EGFR-scores**	**Distant metastases and none-lymph node metastases, EGFR-scores (n=44)**			

	**0**	**1+**	**2+**	**3+**

0	3	0	2	0
1+	1	6	4	0
2+	0	2	9	0
3+	2	1	2	12

**TABLE 3.** EGFR and HER2 scores for all analyzed primary urinary bladder tumors (upper part) and corresponding metastases (lower part)

**Primary tumors EGFR-scores**	**Primary tumors, HER2-scores (n=72)**			

	**0**	**1+**	**2+**	**3+**

0	0	1	2	5
1+	1	0	3	9
2+	4	0	1	13
3+	3	3	12	15

**Metastases EGFR-scores**	**Metastases, HER2-scores (n=72)**			

	**0**	**1+**	**2+**	**3+**

0	2	0	3	5
1+	3	2	0	7
2+	5	3	4	11
3+	3	1	9	14

**TABLE 4.** Summary of relations between positive (2+ and 3+) and negative (0 and 1+) expressions of EGFR in primary tumors and metastases (upper part), between EGFR and HER2 in primary tumors (middle part) and between EGFR and HER2 in metastases (lower part) (n=72)

**EGFR status primary tumors**	**EGFR status metastases**	**Number and % cases**

positive	positive	44 (61%)
positive	negative	7 (10%)
negative	positive	6 (8%)
negative	negative	15 (21%)

**EGFR status primary tumors**	**HER2 status primary tumors**	**Number and % cases**

positive	positive	41 (57%)
positive	negative	10 (14%)
negative	positive	19 (26%)
negative	negative	2 (3%)

**EGFR status metastases**	**HER2 status metastases**	**Number and % cases**

positive	positive	38 (53%)
positive	negative	12 (17%)
negative	positive	15 (21%)
negative	negative	7 (10%)
